# Spectroscopic Evidence of Edge‐Localized States in an Antiferromagnet Topological Insulator NdBi

**DOI:** 10.1002/advs.202522116

**Published:** 2026-01-14

**Authors:** Avior Almoalem, Rebecca Chan, Brinda Kuthanazhi, Juan Schmidt, Jose A. Moreno, Hermann Suderow, Paul Canfield, Taylor L. Hughes, Vidya Madhavan

**Affiliations:** ^1^ Department of Physics and Materials Research Laboratory Grainger College of Engineering University of Illinois at Urbana‐Champaign Urbana IL USA; ^2^ Anthony J. Leggett Institute For Condensed Matter Theory University of Illinois Urbana IL USA; ^3^ Ames Laboratory Ames IA USA; ^4^ Department of Physics and Astronomy Iowa State University Ames IA USA; ^5^ Laboratorio De Bajas Temperaturas y Altos Campos Magnéticos Unidad Asociada UAM‐CSIC Departamento De Fisica de La Materia Condensada Instituto Nicolas Cabrera and IFIMAC Universidad Autonoma De Madrid Madrid Spain

**Keywords:** antiferromagnetic topological insulators, magnetism, monopnictides, NdBi, quasi‐particle interference, scanning tunneling spectroscopy/microscopy, surface states, topological insulators

## Abstract

Materials exhibiting non‐trivial topology and magnetism hold the promise of hosting 1D chiral edge states, which can carry dissipationless currents and, when proximitized to a superconductor, develop Majorana modes. However, persistent materials challenges arising from magnetic and electronic disorder have hindered the realization and measurement of these states, and limited the observation of the Quantum Anomalous Hall effect to low temperatures. Here we study the topological antiferromagnet NdBi, which belongs to a new class of magnetic topological materials, i.e., rare earth monopnictides. These binary topological compounds with intrinsic magnetism are not plagued by the same materials issues and may potentially offer a new platform for hosting 1D edge states. By combining spin‐polarized scanning tunneling microscopy (STM) with quasiparticle interference, we successfully identify distinct signatures of the ferromagnetic (FM) and antiferromagnetic (AFM) terminations. Crucially, we demonstrate that step edges on ferromagnetic surfaces, which serve as magnetic domain walls, host well‐defined 1D edge modes that vanish above the Néel temperature. Our findings position NdBi as a promising platform for further explorations of 1D chiral edge modes and future realizations of Majorana states in proximitized rare‐earth mono‐pnictides.

## Graphical abstract

Using spin‐polarized scanning tunneling microscopy (STM) in conjunction with quasiparticle interference, we successfully identify distinct signatures of the ferromagnetic and antiferromagnetic terminations in NdBi, a rare‐earth monopnictide exhibiting non‐trivial topology and magnetism. We demonstrate that step edges on ferromagnetic surfaces host well‐defined 1D edge modes that vanish above the Néel temperature and on the antiferromagnetic surface.

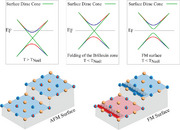

## 1 Introduction

A time‐reversal invariant 3D topological insulator (TI) that develops antiferromagnetism can remain topological, i.e., with a quantized magneto‐electric polarizability and surface states, if the combined symmetries of time‐reversal and translation are preserved [1]. Interestingly, theory shows that ferromagnetic surface terminations of the AFM order will open gaps in the surface Dirac cones and can host gapless chiral modes on magnetic domain walls [2, 3, 4, 5, 6, 7, 8]. These chiral domain‐wall modes serve as a key manifestation of exotic quantum phenomena, including axion electrodynamics and the quantum anomalous Hall effect, and they can generate chiral Majorana fermions when brought into proximity with a superconductor [9, 10, 11, 12, 13, 14, 15, 16, 17, 18, 19, 20, 21, 22, 23].

Rare‐earth monopnictides offer an ideal platform for exploring the interplay between magnetism and topological band structure [24]. Their cubic crystal structure protects the bulk Dirac band crossings [25, 26], and when coupled with the magnetic ground state, these materials can host various electronic phases. A key characteristic of such systems is that the exact magnetic texture is often complex [26, 27, 28, 29, 30, 31, 32, 33, 34, 35, 36]. This inherent flexibility in the spin configuration offers the potential for novel electronic outcomes, ranging from magnetic Weyl semimetals to Type I and Type II AFM materials [26, 27, 28, 29, 30, 31, 32, 33, 34, 35, 36].

A prominent example is NdBi, whose bulk is known to be topologically non‐trivial. This rare‐earth monopnictide features topological Dirac surface states alongside an antiferromagnetic ground state [25, 26, 27, 28, 29, 30], very similar to a canonical AFM‐TI. Most Angle‐Resolved Photoemission Spectroscopy (ARPES) studies [27, 28, 29, 30, 31] have concentrated on what is believed to be the AFM surface, based on Density Functional Theory (DFT) calculations [28, 29, 30]. These studies revealed a unique C_2_‐symmetric surface state with an unusual spin texture below the Néel temperature (T_Néel_) [25, 26, 27, 28, 29, 30]. Recent nano‐ARPES studies have shown that the surface states disappear in certain domains, which were attributed to the presence of a ferromagnetic (FM) surface termination where the spins are ferromagnetically aligned [28, 29]. However, this identification is unproven as more complex spin textures can exist, as in other rare‐earth monopnictides [26, 32, 33, 34, 35, 36]. Consequently, a direct, definitive proof for the existence of gapped surface Dirac cones and 1D edge modes associated with FM terminations is still lacking. Moreover, DFT suggests that the band inversion in the FM domain occurs below and above the Fermi level [26]. This raises critical questions concerning the relevant energy levels for the existence of a chiral edge state, as predicted by theory, and the effect that trivial bands in proximity to the topological bands would have on such a state [25, 26, 29, 37]. This lack of comprehensive understanding of the sample's diverse electronic phenomena presents a significant challenge for developing a complete theoretical framework for these materials [25, 26, 27, 28, 29, 30], especially in efforts to accurately capture the essential physics governing these systems [26].

In this work, we present spin‐polarized scanning tunneling spectroscopy and microscopy (SP‐STS/STM) measurements. These techniques are crucial as they enable us to establish a local and direct correlation between the surface spin texture and the material's electronic properties [35, 36]. Critically, we provide the direct proof of the existence of the 1D localized edge state [1, 2, 3, 4, 5, 6, 7, 8, 38]. This 1D state is uniquely observed in the magnetically ordered phase and appears only at a specific termination: the ferromagnetic (FM) surface. This one‐to‐one correlation between the termination type and the existence of the edge state aligns perfectly with the theory of the AFM topological insulators, which predicts it to be a chiral edge state.

**FIGURE 1:**
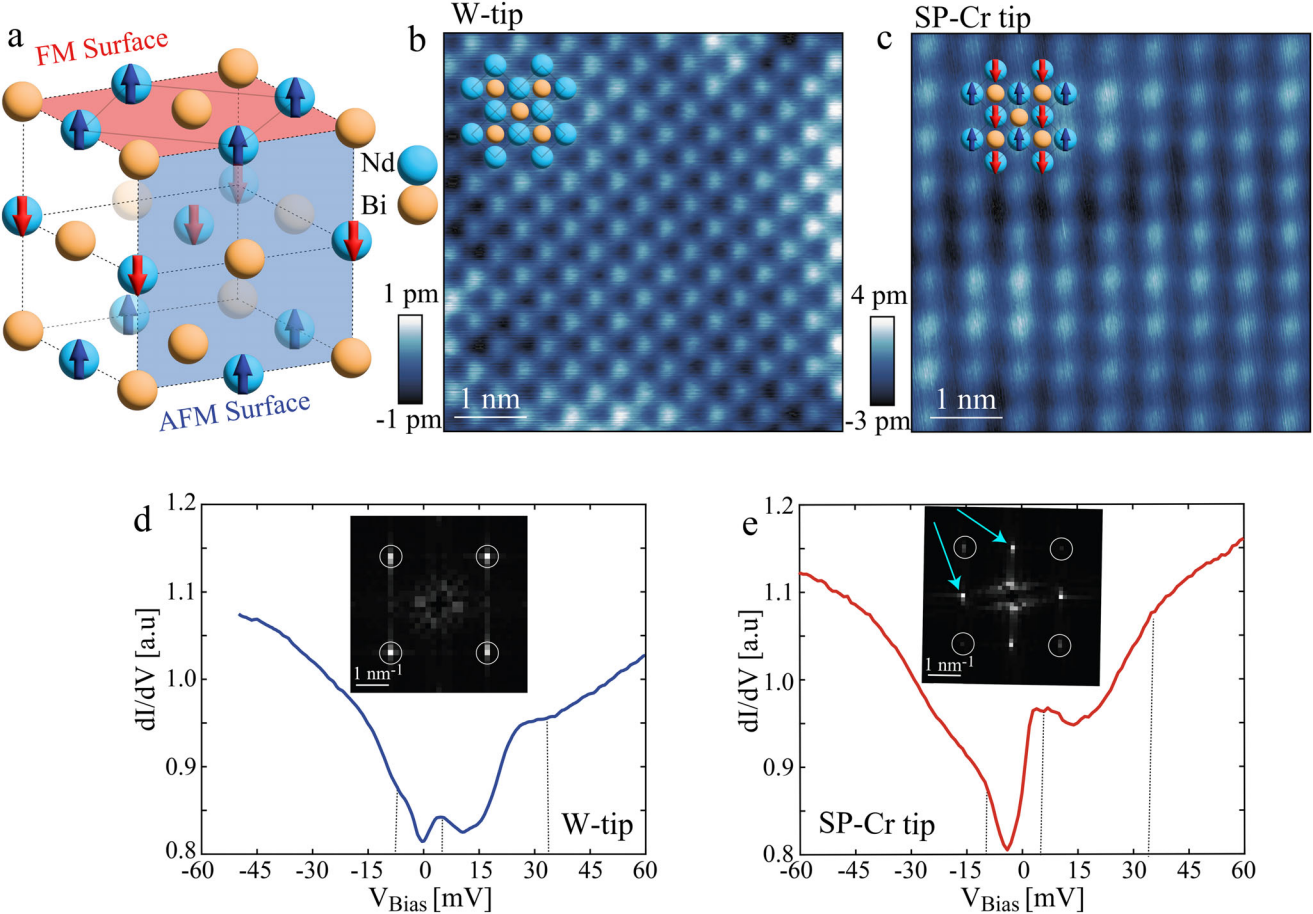
NdBi crystal structure, STM topography, and spectra. (a) Crystal and magnetic structure of NdBi with the Bi atoms in orange and Nd in blue. The crystal has a rock‐salt structure with the local moments residing on the Nd atoms. (b) Topography of the NdBi surface with a W tip, V_s_ = 50 meV, I_t_ = 700 pA. (c) Topography of the NdBi surface with a spin‐polarized Cr tip, V_s_ = 25 meV, I_t_ = 250 pA. (d) Averaged spectra taken on the same locations as b with a W tip, V_s_ = 60 meV, I_t_ = 360 pA. Inset: FFT of the topography in a with the Bragg peaks (white circles) matching a translation vector with a size of 0.46 mn. (e) Averaged spectra acquired on the same location as c using a Cr tip, V_s_ = −70 meV, I_t_ = 250 pA. Inset: FFT of the topography in c showing the AFM peaks (cyan arrows). The two spectra taken using the W and Cr tips give the same overall features, as shown by the dashed black lines.

We begin with a description of the known properties of NdBi. The paramagnetic phase of NdBi hosts topologically non‐trivial bands characterized by a Fu‐Kane topological index of (*ν_0_, ν_1_, ν_2_, ν_3_*) = (1,0,0,0) [25, 26, 27, 28, 29, 30], which indicates a strong topological insulator (STI). Band inversion occurs between the Bi‐6p and Nd‐5d orbitals along the *Γ‐X* line [28, 29]. NdBi has a rock‐salt crystal structure, which, below the Néel temperature of 24K (Figure S1), is a layered AFM [25, 26, 27, 28, 29, 30] as shown in Figure 1a. In the AFM phase, time‐reversal and translational symmetries are individually broken; however, their combination remains preserved in the bulk [1, 15, 16]. For a given crystal, both FM and AFM surface terminations are realized as different facets of the same bulk spin structure (Figure 1a). In fact, within one single crystal, the material may host different bulk magnetic domains with the surface termination being either FM or AFM.

## 2 Results

As previously discussed, NdBi is a bulk antiferromagnet in which Nd and Bi atoms adopt a cubic, rock‐salt crystal structure (Figure 1a). In the magnetically ordered phase, within one crystal, one can have multiple magnetic domains where the surface terminations are either AFM or FM. To carry out STM studies, NdBi samples were cleaved at ∼300K and at pressures lower than 5E‐10 torr, before being directly inserted into the STM head held at 1.9K. To understand the magnetic structure and correlate it with the surface electronic structure, STM data were acquired both with spin‐polarized Cr tips and unpolarized W‐tips, at *B* = 0T. Cr tips were characterized on the antiferromagnet FeTe before measuring NdBi. The tips show a polarization reversal, observed as a phase shift of the magnetic contrast on NdBi, confirming that they are polarized (Figure S2). Topography obtained with W‐tips (Figure 1b) reveals surfaces consistent with imaging the non‐magnetic NdBi unit cell, where only one species (either Nd or Bi) is visible. However, images obtained with the Cr tip reveal two different surface terminations, one with the same periodicity as the W‐tip, and the other with a larger periodicity where the distances between the atoms are √2 times larger (Figure 1c).

### 2.1 AFM Surface and QPI Signature

We first focus on surfaces that display a larger unit cell with spin‐polarized tips. This domain comprises the majority of the sample's surface. In the tunneling process with a spin‐polarized tip, the matrix element is suppressed for spins anti‐parallel to the tip apex‐spin, and enhanced for parallel spins [35, 36, 39, 40, 41]. Consequently, the tunnel current from surface atoms is either amplified or diminished according to their spin orientation, therefore enhancing the contrast between the different spin projections in topography scans. Thus, the images with the larger unit cell correspond to an antiferromagnetic configuration in which each spin is surrounded by anti‐parallel neighbors (AFM surface). The spin structure can also be seen in the corresponding fast Fourier transform (FFT) of the AFM surfaces obtained with Cr tips, which reveal characteristic peaks at wavevector *q* = 1/√2 and a 45° rotation relative to the Bragg peaks seen in the FFT of the W tip images, as shown in the insets to Figure 1d,e. The real‐space and momentum‐space periodicity is as expected from the AFM unit cell, as shown in Figure 1c. While the surfaces may look different with W‐ and Cr‐tips, the *dI/dV* spectra show similar overall features: a dip near *E_F_* as well as peak‐like features at similar energies ∼ −10, 5, and 30 mV (Figure 1d,e).

**FIGURE 2:**
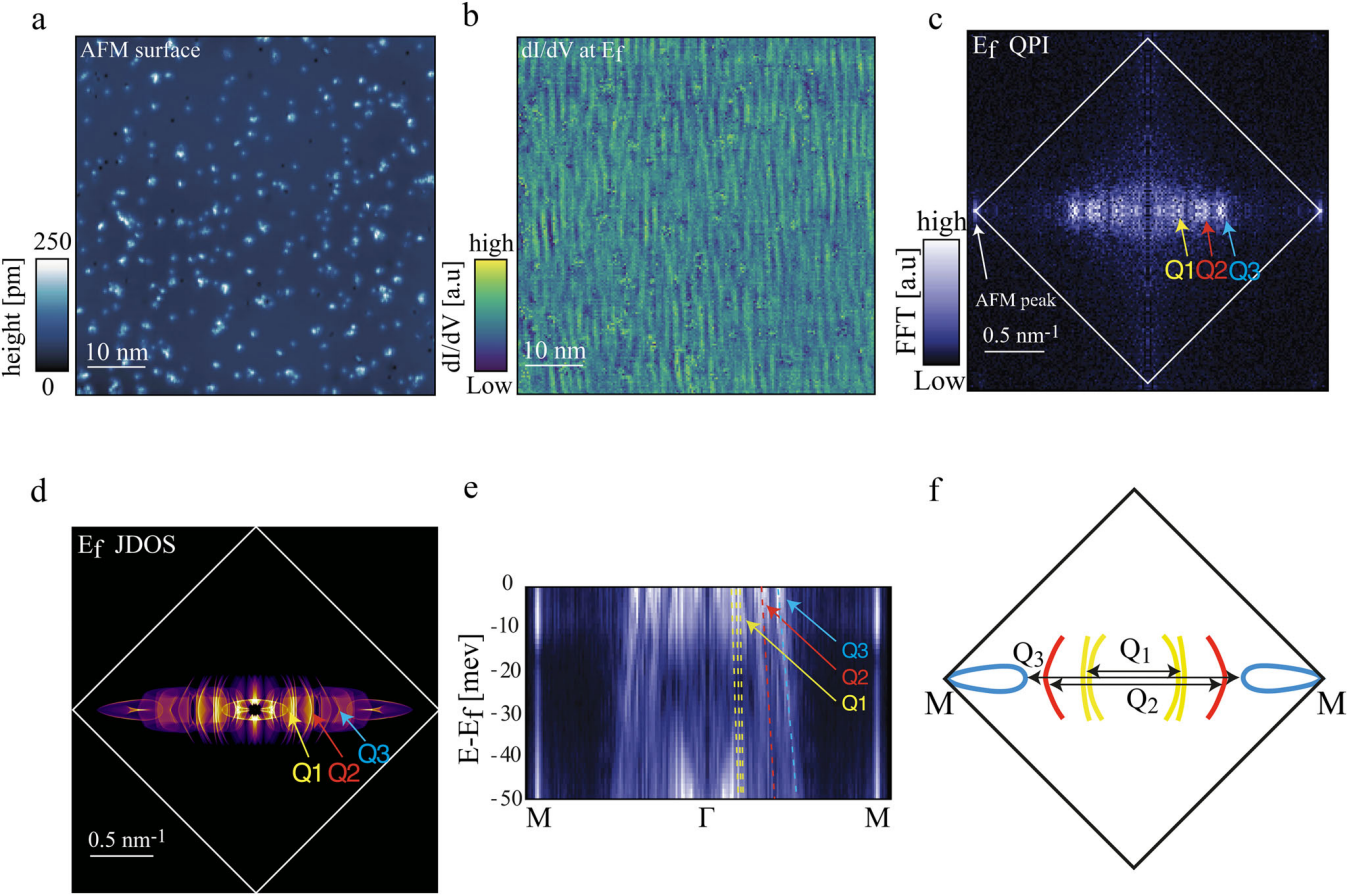
QPI data on the AFM surface. (a) Topography of an AFM surface with scattered adatoms, where the dI/dV maps were obtained. V_s_ = −50 meV, I_t_ = 160 pA. (b) E = E_F_ slice of the dI/dV map taken on the region shown in (a), showing the quasiparticles interference patterns with clear C2 symmetry. I_t_ = 160 pA, V_s_  = −50 meV, V_mod_  = 6 meV, f  = 907.5 Hz, (c) FFT of the dI/dV slice in (b), showing the wave vectors Q1, Q2, Q3 corresponding to scattering of the quasiparticles from the electron‐like surface state, hole‐like Fermi arcs, and additional surface states around the Γ point. The white square represents the Brillouin zone in the non‐magnetic state, with the AFM peak at the M point. (d) Calculated FFT of the QPI patterns of the surface states and Fermi arcs in a single AFM domain obtained by taking the autocorrelation of the Fermi surface shown in (f). The white square represents the Brillouin zone. (e) QPI dispersion acquired from the map taken along the Γ‐M line, parallel to the Q vectors. The dispersion is visible and compared within a 50 meV energy window of the negative bias side. The dashed lines represent the ARPES dispersion from ref [27]. (f) Cartoon of the Fermi surface from ref [27]. The Fermi arcs are shown as yellow and red lines, while the electron‐like surface states are shown using a blue line, in the Brillouin zone of the paramagnetic phase, with the M point marked. The main scattering vectors are labelled Q_1_, Q_2_, and Q_3,_ respectively. Q1 represents the scattering within the surface state encircling the Γ point. Q2 corresponds to scattering between the hole‐like Fermi arcs. Q3 is the scattering between the electron‐like surface states. Data was acquired using a Cr tip.

To reveal the relationship between the magnetic structure and the emergent electronic phenomena, we investigate the electronic structure of the AFM surface via quasi‐particle interference (QPI) measurements. In general, *dI/dV* maps provide insight into spatial variations of the local density of states (LDOS), which capture the interference patterns generated by the scattering of quasi‐2D bulk states, surface states, and Fermi arcs [42, 43, 44, 45, 46, 47]. By applying Fourier analysis to these maps, the contributing scattering processes can be decomposed according to their respective momentum transfers. A large‐area topographic‐image of an AFM surface featuring adatoms (bright spots) and vacancies (dark spots) is shown in Figure 2a. Lattice defects, originating from the adatoms or vacancies, can induce quasi‐particle scattering events. As shown in Figure 2b, these events generate C_2_‐symmetric wave‐like patterns. Consequently, the FFT of the *dI/dV* maps reveals wave vectors along the *Γ–M* direction (Figure 2c). We note here that all QPI data shown in the paper were symmetrized according to the intrinsic C_2_ symmetry of the data. The corresponding unsymmetrized data are provided in Figures S3 and S4.

The momentum‐space location and C_2_‐symmetric structure of the QPI are reminiscent of the non‐topological surface states measured by ARPES, which exhibit C_2_ symmetry [27, 28, 29, 30, 31]. DFT calculations [29] indicate these C_2_ symmetric features are associated with the AFM surface. To confirm the origins of the measured QPI, we use the ARPES‐derived C_2_ symmetric bands to simulate the expected QPI signal by calculating the joint density of states (JDOS), using the autocorrelation of the Fermi surface (Figure 2d), which we then compare with our QPI data. Since QPI signals are predominantly derived from 2D (or quasi‐2D) electronic states, we use the emergent electron‐ and hole‐like surface states, as well as Fermi arcs, observed in a recent ARPES paper [27, 28, 29] (Figure 2f) for the QPI simulation. We note that the topological Dirac surface states are not included here since backscattering is forbidden [48, 49] and they will not generate a QPI signal. For completeness, a similar JDOS calculation, which includes the Dirac cones, is shown in Figure S5. We note that in our simulation of the QPI data using JDOS, we did not make any spin‐selective or matrix element assumptions.

Upon comparing the JDOS shown in Figure 2d with the FFT in Figure 2c, we conclude that the primary scattering processes observed include: (i) scattering from hole‐like surface states around the *Γ* point (Q1), (ii) scattering between hole‐like Fermi arcs (Q2), and (iii) scattering between electron‐like surface states (Q3). The corresponding dispersions are shown in Figure 2e and are consistent with ARPES data from previous studies [27, 28, 29]. The QPI signal weakens considerably for energies below −50 meV and vanishes entirely at −120 meV (Figure S6), which is also consistent with the dispersion characteristics of surface states and Fermi arcs as observed in ARPES studies. Identical QPI patterns are observed using both the Cr and W tip, and the QPI remains consistently aligned along the *Γ–M* direction of the nonmagnetic Brillouin zone (Figure S6). It is noteworthy that the QPI cannot be accurately reproduced without accounting for surface states close to the *Γ* point (Q1), shown in Figure 2f, where the Fermi level contour of the surface states and the corresponding scattering vectors are presented.

To further correlate the AFM phase with the emergent QPI, we conducted temperature‐dependent measurements at 1.9K and 25K, i.e., below and above *T*_Néel_. Both data sets, above and below the transition temperature, were acquired under the same STS conditions and at the exact same location. Within the magnetic phase, strong QPI signals and distinct AFM peaks in the FFT are observed, and upon heating the sample slightly above *T*_Néel_, the AFM peaks disappear as expected (Figure S7). Moreover, signatures of QPI are absent at all energies above *T*_Néel,_ which indicates that the associated electronic states responsible for the QPI are likewise absent. This is fully consistent with ARPES data and DFT, which show that the Fermi arcs and electron‐like surface states as presented in Figure 2f emerge exclusively in the AFM phase [28, 29].

### 2.2 FM Surface and 1D Edge Modes

**FIGURE 3:**
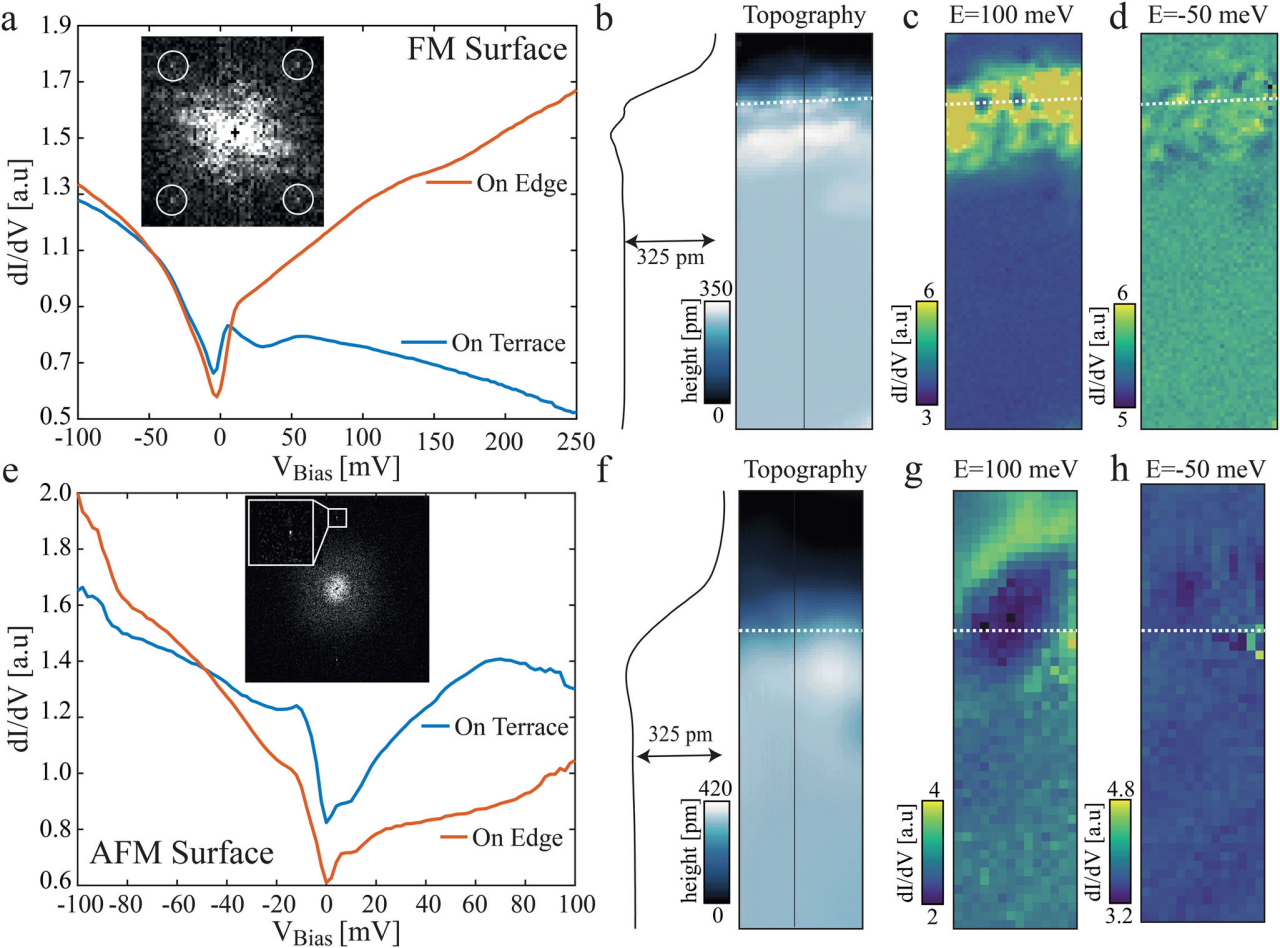
Step Edge spectra and maps on an FM and AFM surface. (a) dI/dV spectra acquired on a terrace and on a step edge of an FM surface. An average taken on the edge (orange curve) shows the increased conductance on the step edge relative to the average taken on the terrace (blue curve). Inset: FFT of the topography taken on the terrace showing no AFM peaks. White circles mark the paramagnetic unit cell Bragg peaks. (b) Topography taken in a region with a step edge and the line profile obtained at the solid vertical black line. V_s_ = −100 meV, I_t_ = 150 pA. (c) dI/dV map at E = 100 meV on the same area showing the edge state, which is localized within 1.5 nm of the edge. (d) dI/dV map at E = −50 meV. Dashed white lines mark the location of the edge in all panels. All scale bars in (b–d) are 1 nm. V_s_ = −100 meV, I_t_ = 150 pA, V_mod_ = 4 meV. (e) Same as (a) for an AFM surface. Inset: FFT of the topography showing AFM peaks. (f–h) Topography and dI/dV maps taken on the edge, with a height of a single layer, as in (b). There is no enhanced density of states on the edge state in either map, emphasizing the absence of the edge state on the AFM surface. V_s_ = −100 meV, I_t_ = 150 pA, V_mod_ = 4 meV. Dashed lines are in the same location in all panels, to mark the location of the edge. All scale bars in (f–h) are 0.5 nm. Data was acquired using a Cr tip.

We now move on to the surface with the broken time reversal symmetry, i.e., the ferromagnetic surface. As shown in Figure 1a, this surface represents the FM termination of stacked layers of spins in which the spins within each layer are co‐aligned but alternate in orientation between consecutive layers. FM domain walls in NdBi may, in theory, host chiral edge states since the gapped topological surface states may bind chiral modes on such boundaries. The FFT of a topography obtained with the Cr tip on such a surface is characterized by a noticeable lack of AFM peaks (Figure 3a inset). This essentially means that the tunneling matrix element is the same for every Nd atom on the surface with a spin‐polarized tip, which indicates uniform magnetism, which is as expected for an FM region. Fourier transforms of *dI/dV* maps on this surface are uniformly featureless (Figure S8). The lack of both AFM ordering and QPI is consistent with DFT calculations for the FM surfaces, which do not exhibit the surface states or Fermi arcs that were the main contributors to our QPI signal on the AFM surface [29]. We use these features as a fingerprint of FM surfaces.

**FIGURE 4:**
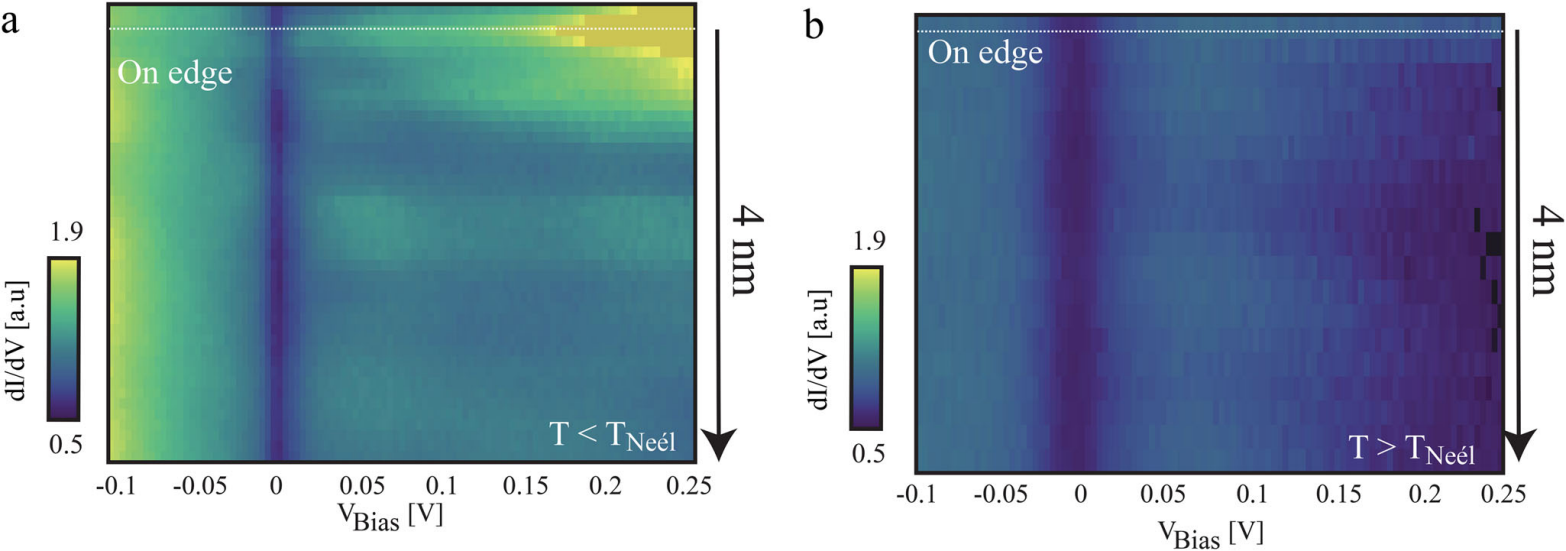
Presence and absence of the edge state below and above *T*_Néel_. (a) *dI/dV* spectra acquired along the line shown in Figure S7, a clean edge on a FM surface, below *T*_Néel_. The 1D chiral edge state is shown as an increase in conductance, localized on the edge (marked by a horizontal dashed line). As shown in Figure 3, the increase in conductance is in the positive bias energies, above the Fermi level. (b) Same as above, *T*_Néel_. The enhanced conductance is gone, and the spectra are similar on and off the edge. Data were acquired along the line marked in the inset of Figure S7. *V_s_* = −100 meV, *I_t_* = 150 pA, *V_mod_*  = 4 meV. Data was acquired using a Cr tip.

To investigate the existence of 1D edge states, we examine step edges (Figure 3b) as was done in a recent study of MnBi_2_Te_4_ [38]. Odd step edges on the FM domains (Figure 4e) separate terraces with opposite spin ordering and therefore act as magnetic domain walls [1, 2, 3, 4, 5, 6, 7, 8]. The step edge height shown in Figure 3b corresponds to an odd number of layers and thus separates layers with opposite spins and effectively acts as a magnetic domain wall. Figure 3a shows *dI/dV* spectra measured on the terrace and near the step edge. The density of states at the step edge is larger than the terrace for a wide range of energies above the Fermi energy (E_F_). This enhanced density of states is clearly visible in dI/dV maps measured in this area (Figure 3c), which reveal a ∼15 Å wide region along the edge with enhanced conductance. We fit the conductance as a function of distance from the edge to a Gaussian decay function, and compared the penetration depth of the edge state to the known relation from the Bernevig‐Hughes‐Zhang model ξ  =  ℏ*v_F_*/Δ [49, 50, 51]. Using published ARPES data [28] for the Dirc cone at the gamma point beneath the Fermi level we estimate the expected deca length scale, $v_{F}=\frac{1}{\hbar }\;\frac{dE}{dK}=\frac{1}{\hbar }\;\frac{170\;\mathrm{meV}}{0.072\;{Å}^{-1}}$=  2360 meVÅ, Δ  =  170meV, we get ξ  =  14Å  =  1.4 nm. This is in an excellent agreement with our results (Figure S9). As further elaborated on in the discussion section, the fact that the density of states enhancement occurs at energies above E_F_ is expected according to previous DFT calculations [27]. The presence of these edge states was reproduced on step edges on multiple samples studied with both Cr tips (Figure S10) and W‐tips (Figure S11).

As an additional confirmation, we examine odd step edges on the AFM surface. The FM surface, with staggered magnetization on the surface, results in gapped surface Dirac cones and ultimately in the formation of the edge state. Following this argument, the edge state should not exist on the step edge of an AFM‐surface [1]. We identify and measure a suitable AFM step edge in the same area where the FFT reveals strong AFM peaks, as shown in Figure 3e,f. The *dI/dV* map on an AFM surface step taken under the same conditions as the FM terrace (Figure 3g,h), reveals that the edge shows no enhancement and in fact shows a decrease in conductance, Figure S8. This is also reflected in spectra obtained over a broader energy range, as illustrated in Figure S12.

Importantly, the existence of the edge state on the step edges of the FM surface is correlated with the onset of the magnetic phase. We measured spectra on the FM surface at different temperatures, below and above *T*_Néel_, as shown in Figure 4. In the paramagnetic phase (*T>T*_Néel_), the edge spectra show no enhancement over the bulk, i.e., the increase in density of states characterizing the edge state is notably absent. Similar spectra were observed at different positions along a line perpendicular to the edge. These measurements demonstrate that the edge state exists only below the *T*_Néel_ and disappears above it, along with magnetism.

## 3 Discussion

Our studies establish the correlation between the surface type (ferro‐ or antiferro‐magnetic) and boundary states in NdBi. For surfaces with an AFM component, the presence of electron‐like surface states and Fermi arcs generates a strong C_2_‐symmetric QPI signal with a dispersion that is consistent with ARPES studies. Considering an AFM surface as shown in Figure 1a, for every given Nd atom, the nearest neighbor Nd atom possesses an AFM‐aligned spin, while the next nearest neighbor possesses a parallel spin. This in turn expands and rotates by 45°, the unit cell on the surface, thus preserving C_4_ symmetry seen through Bragg peaks in FFT data. Moreover, as DFT suggests for such AFM surfaces, the spin direction in real space dictates the direction in which the C_2_ symmetric surface states and Fermi arcs emerge [29, 30]. In our study, the surface states are aligned with one pair of AFM Bragg peaks, thus suggesting the spins on every Nd atom are aligned along a specific lattice unit vector. Although the origin of such C_2_ symmetric surface states is still not known [26, 27, 28, 29, 30, 31, 32, 33, 34], it naturally explains our QPI C_2_ symmetry. Apart from the qualitative agreement between the QPI symmetry and the surface states measured by ARPES, the dispersion of the STM scattering vectors agrees well with the ARPES dispersion [27, 28].

A recent spin‐polarized ARPES study, accompanied by DFT results, shows that the non‐topological surface states [29, 30] have a complex spin texture. For spin‐momentum locking, such as the case of topological Dirac surface cones, the opposite spin alignment of states with opposite momenta eliminates the scattering matrix‐element for non‐magnetic scatterers [48]. On the other hand, for a regular trivial surface state or a more complex spin texture with the spin projection existing in all directions, the matrix element of such a scattering process has a finite value. This allows for scattering and thus gives rise to the Q_i_ (*i* = 1,2,3) scattering vectors seen in our study. This observation suggests that the surface states, which are associated with the AFM phase as observed in our QPI data, possess a spin texture that does not forbid backscattering, similar to QPI measured in Weyl semimetals [42, 44, 46, 47]. We reiterate that the QPI data do not capture the three massless Dirac surface cones that exist in both the AFM and paramagnetic phases—one at the Brillouin zone center (*Γ*) and two at the *M* points [27, 28]. The lack of a QPI signature from the Dirac cones is attributable to their topological nature (spin‐momentum locking), which prohibits backscattering [48, 49]. QPI measurements on other TI's, such as Bi_1‐x_Sb_x,_ show that states with opposite momentum and opposite spin configuration cannot scatter from one another [48]. This QPI disappears above *T*_Néel,_ which correlates it with the magnetic ground state. The second kind of surface, where only the paramagnetic unit cell is seen with the same spin‐polarized tip, can thus be designated as the FM surface. The lack of QPI on FM surfaces reveals the correlation between the surface spin texture and the electronic properties, providing evidence that the rare‐earth monopnictide NdBi is a type I AFM [25, 26, 27, 28, 29, 30, 31, 32, 33, 34, 35, 36].

According to theory, in some members of the rare‐earth monopnictides family with a strong TI index for the non‐trivial bands in the band structure, the FM surface supports a chiral edge state [1]. In our data, we observe 1D edge modes spanning a large energy range on the FM terminations. Although enhanced 1D conductance along step edges can result from more trivial effects such as dangling bonds or strong edge‐induced scattering [37], the lack of 1D edge states on AFM step edges or above $T_{\mathrm{Neel}}$ rules out this interpretation. The AFM surface step edges and FM surface step edges have identical crystal structures and differ only in the spin structure, so any dangling bond states should be observed on both edges. Moreover, dangling bonds do not alter with small temperature changes (∼24K) and therefore should exist above the transition temperature. Another suggested explanation is the existence of hinge modes due to the Dirac semi‐metal [26] phase. However, such hinge modes should also exist in the paramagnetic phase, contrary to our data. The 1D edge state we observe most likely arises from physics analogous to that of an antiferromagnetic topological insulator, where theory predicts a chiral character. This behavior is consistent with what is expected at step edges in AFM topological insulators.

We note that the data on the FM and AFM surfaces were acquired with the same Cr tip, emphasizing the correlation between the FM surface in the magnetic phase and the appearance of the edge state. Equally important, we emphasize that tunneling spectra acquired on adatoms exhibit no conductance enhancement (see Figure S13) over a large range of energies. Consequently, the observed edge state cannot be attributed to a trivial effect arising from surface adatoms.

**FIGURE 5:**
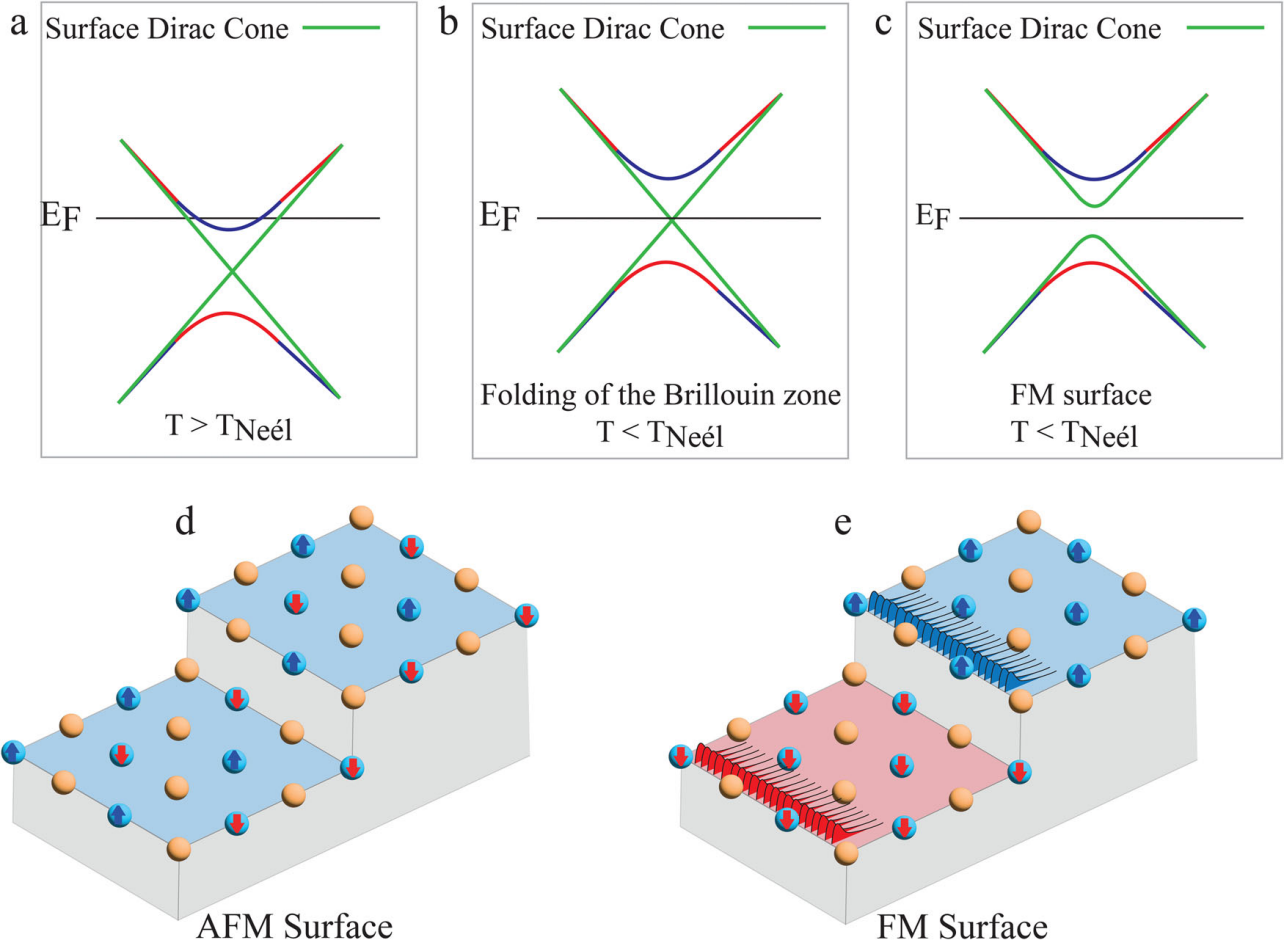
Cartoon figure of bulk topological band structure, surface state, and formation of edge states. (a) Above T_Néel,_ the material is a strong topological insulator with the band inversion and Dirac point below E_F_ according to DFT calculations presented in Ref. [29]. (b) After the system is cooled into the AFM phase, the doubling of the unit cell in k_z_ pushes the band inversion to a higher energy (along Γ‐Z and Γ‐M lines). (c) On the FM surface, the Dirac cones are gapped due to the magnetic order and create edge states. (d) and e Step edges of the AFM and FM surfaces, respectively. Odd step edges on the FM surfaces act like domain walls and trap chiral edge states.

We now discuss the fact that the edge state is seen in our measurements most clearly at energies above E_F._ As we describe below, this can be attributed to the increased bulk density of states below E_F_. To understand this, let's consider the effects of the band folding in the magnetic phase caused by increasing the unit cell size in the z direction. As a result of the band folding, the band inversion along the *Γ‐Z* and *Γ‐M* lines shifts upward in energy. The band inversion along the *Γ‐M* line spans from negative to positive energies, while the *Γ‐Z* band inversion is completely above the Fermi level (see Figure S14) as predicted by DFT results [29]. The magnetization on the FM surface ultimately produces a gapped Dirac cone with a gap of several hundred meV as seen in surface spectral function calculations [28, 29] and illustrated in Figure 5. This suggests that the edge state should appear both below and above E_F_. However, as can be clearly seen from the *dI/dV* spectra in Figure 3a,e, the density of states rises sharply below E_F,_ which obscures the edge state below *E_F_*, Figure 3d. This scenario is reinforced by the fact that spectra acquired on the AFM surface show no clear changes at *E* = −50 meV, where the surface state starts dispersing, indicating that the signal is also swamped by the bulk density of states.

By demonstrating the presence of both AFM and FM domains through spin‐polarized STM, our data offer definitive confirmation of the spin structure in NdBi and extend the AFM topological‐insulator picture to systems containing a mixture of trivial and non‐trivial bands. This is similar to chiral step‐edge states observed in the Weyl semimetal phase of Co_3_Sn_2_S_2,_ where the modes are tightly localized on step edges [37]. Unlike Co_3_Sn_2_S_2,_ however, where only certain rarely seen terminations show edge modes, rare‐earth monopnictides have the advantage that any FM domain should show these 1D states. Importantly, since the FM surfaces in these materials host gapped Dirac cones and no other surface states or Fermi arcs, the edge states remain isolated and well defined. Our work thus sets the stage for further investigations of 1D edge states, and their applications as building blocks for Majorana quasiparticles in proximitized rare‐earth monopnictides and superconductors.

## 4 Materials and Methods

Single crystals of NdBi were grown out of In flux. Elements with an initial composition of Nd_6_Bi_6_In_94_ were placed in a 2 mL alumina fritted Canfield Crucible Set [52, 53] and sealed in a fused silica tube under a partial pressure of Argon. The prepared ampules were heated up to 1150°C over 5 h and held there for 2 h. This was followed by a slow cooling to 700°C over 120 h and decanting of the excess flux using a centrifuge [54]. The cubic crystals obtained were stored and handled in a glovebox under a nitrogen atmosphere. STM measurements were performed using a Unisoku STM at an instrument temperature of 1.9K (unless otherwise specified) using chemically etched and annealed tungsten and chromium tips. Spectra were acquired using a standard lock‐in technique at a frequency of 907.5 Hz.

## Author Contributions

A.A. and V.M. conceived the experiments. The single crystals were provided by B.K., J.S., J.A.M. and P.C. A.A. obtained the STM data. A.A. and V.M. performed the analysis, and R.C. and T.L.H. provided the theoretical input on the interpretation of the data. A.A., V.M., and T.L.H. wrote the paper with input from all authors.

## Conflicts of Interest

The authors declare no conflicts of interest.

## Supporting information




**Supporting File**: advs73791‐sup‐0001‐SuppMat.docx.

## Data Availability

The data that support the findings of this study are provided in the main text and the Supporting Information. The original data is available from the corresponding author upon request.
