# Supplementary material for: Spectroscopic Evidence of Edge‐Localized States in an Antiferromagnet Topological Insulator NdBi
Source: Adv Sci (Weinh). 2026 Jan 14;13(30):e22116. doi: 10.1002/advs.202522116 (PMC13248813; doi:10.1002/advs.202522116)
Supplement: Supplementary file 1 — Supporting File: advs73791‐sup‐0001‐SuppMat.docx. [file ADVS-13-e22116-s001.docx]

Supplementary Materials for

Spectroscopic Evidence of Edge-Localized States in an Antiferromagnet Topological Insulator NdBi

Avior Almoalem^1*^, Rebecca Chan^1,2^, Brinda Kuthanazhi^3,4^, Juan Schmidt^3,4^, Jose A. Moreno^3,5^, Hermann Suderow^5^, Paul Canfield^3,4^, Taylor L. hughes^1,2^ & Vidya Madhavan^1^

1. Department of Physics and Materials Research Laboratory, Grainger College of Engineering, University of Illinois at Urbana-Champaign, Urbana, IL, USA
2. Anthony J. Leggett Institute for Condensed Matter Theory, University of Illinois, Urbana, IL, USA
3. Ames Laboratory, Ames, Iowa, USA
4. Department of Physics and Astronomy, Iowa State University, Ames, IA, USA
5. Laboratorio de Bajas Temperaturas y Altos Campos Magnéticos, Unidad Asociada UAM-CSIC, Departamento de Fisica de la Materia Condensada, Instituto Nicolas Cabrera and IFIMAC, Universidad Autonoma de Madrid, E-28049 Madrid, Spain

**Sample characterization:**


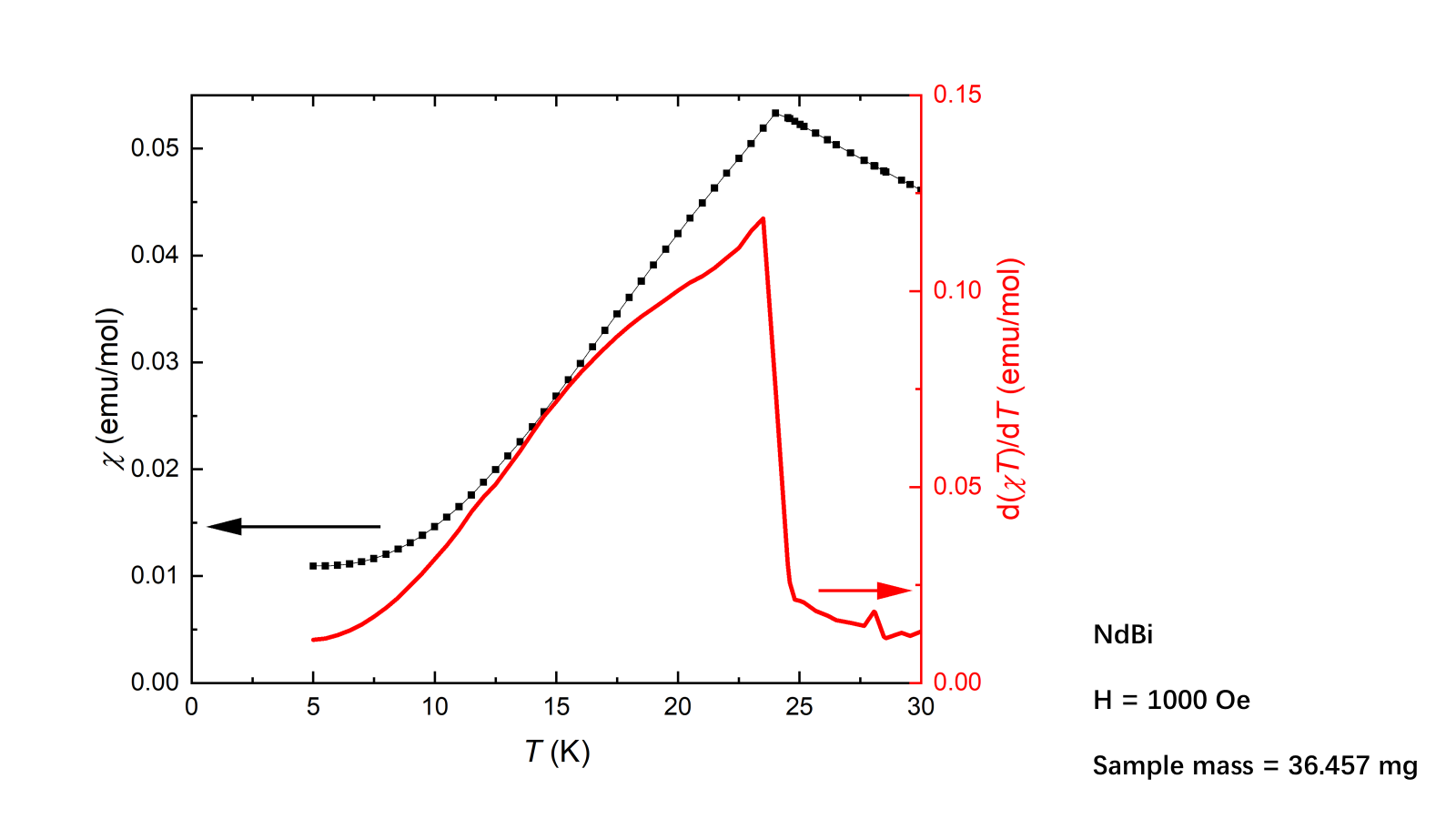


Figure S1 – Magnetic Susceptibility measurements of the samples used in the paper. Data was taken between 5 and 30K, at applied magnetic field of B=0.1T. A clear change in the sample susceptibility is observed at 24K signaling the onset of the magnetic phase.

**Cr tip characterization:**


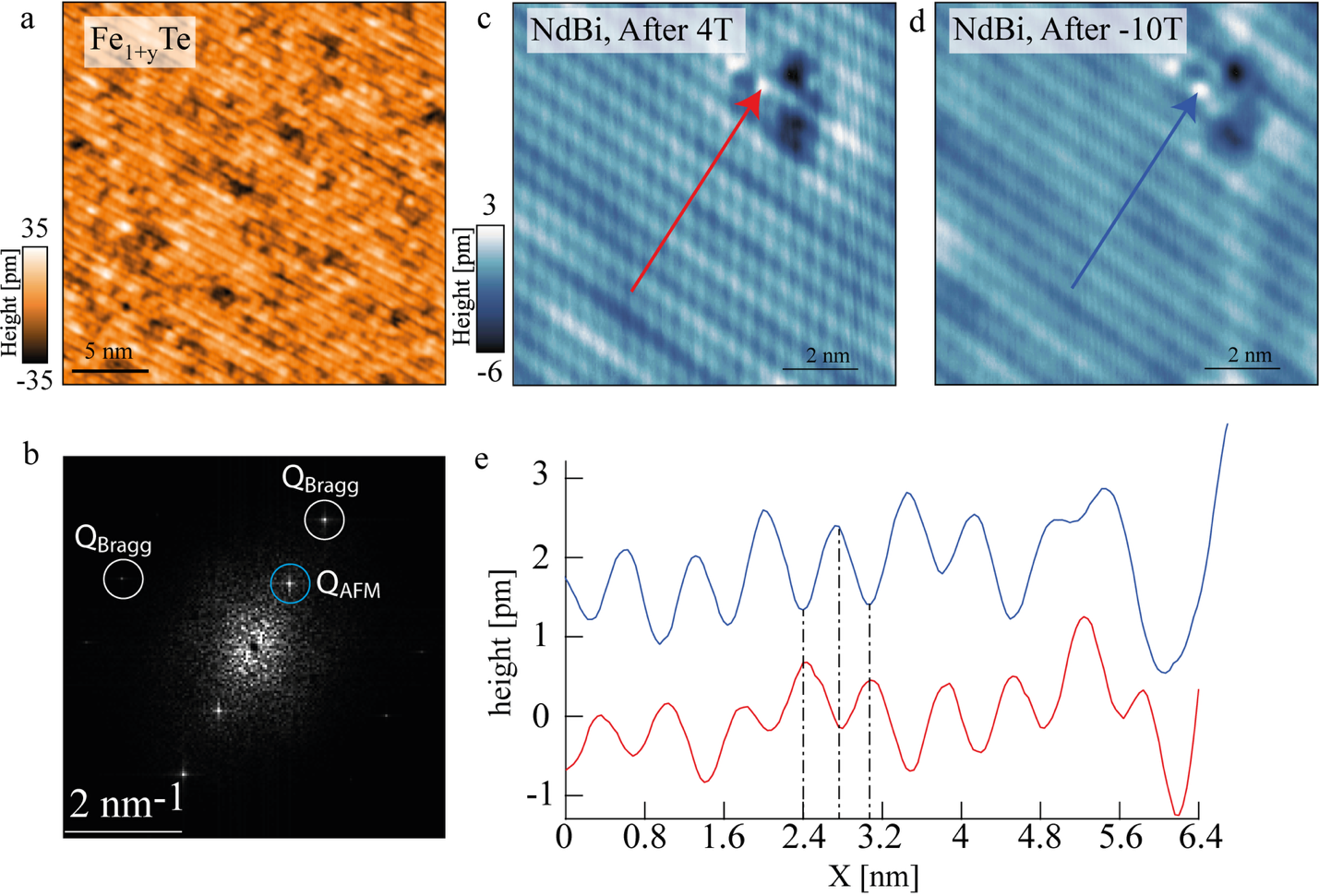


Figure S2 –Characterizing the Cr tip. **a**. Topography scan acquired on a Fe_1+y_Te crystal with the Cr tip used for NdBi measurements. V_s_ = -30 meV, I_t_ = 300 pA. **b** FFT of the scan presented in **a** showing both Bragg peaks (white circles) and the AFM stripes peaks (cyan circle) along one of the Bragg peaks and at half of the q vector size, matching the two atomic lattice sites AFM phase in this crystal. **c** Topography scan of NdBi at B=0T acquired after a tip polarization with B = 4T magnetic field. **d** Topography scan of NdBi at B=0T acquired after a tip polarization with B = -10T magnetic field. Both **c** and **d** were taken at the same location. The two bright and dark spots are used to align the two scans, before and after polarizing the tip, to show the flipping of the spin. **e** A line profile taken on the two scans with the same line length, starting at the same point and ending at the same point (the bright defect). The line profiles give the corrugation on the surface. There is a clear shift of the profile due to flipping of the tip polarization. The AFM signal and the tunability of the tip with magnetic field confirm that the tip is indeed spin polarized. **c, d** V_s_ = -8 meV, I_t_ = 150 pA. Data was acquired using a Cr tip.

We prepare Cr tips on a Fe₁₊ₓTe sample following established procedures [39-41], Supplementary Figure S2. The observed difference in apparent atomic heights, using a Cr tip as opposed to a W tip, is attributed to the spin-selective tunneling process occurring with the Cr tip. A similar effect exists in Fe₁₊ₓTe, where the AFM state of the Fe lattice is resolved despite the exposed surface being the Te layer, Supplementary Figure S2a.

To confirm the spin polarization nature of the Cr tip, we measure the same location, on the NdBi sample, at *B*=0T, once after polarizing the tip at *B*=4T and then after polarizing the tip at *B*= -10T. To overcome a general shift of the sample we located the exact same location using the distinct three defects seen in Supplementary Figure S2c,d. After carefully aligning the two maps according to these three defects, a shift in atom locations is measured, due to the flipping of the spin at the apex of the tip. At first, the tunneling process is mainly into the “up” spins, thus we measure a specific spin orientation. After polarizing the spin at the apex of the tip in the opposite direction, the tunneling is into the “down” spin atoms, thus shifting the scan. The shift is seen in a line cut taken from both scans as seen in Supplementary Figure 2e.

**Additional QPI maps raw (unsymmetrized) data:**


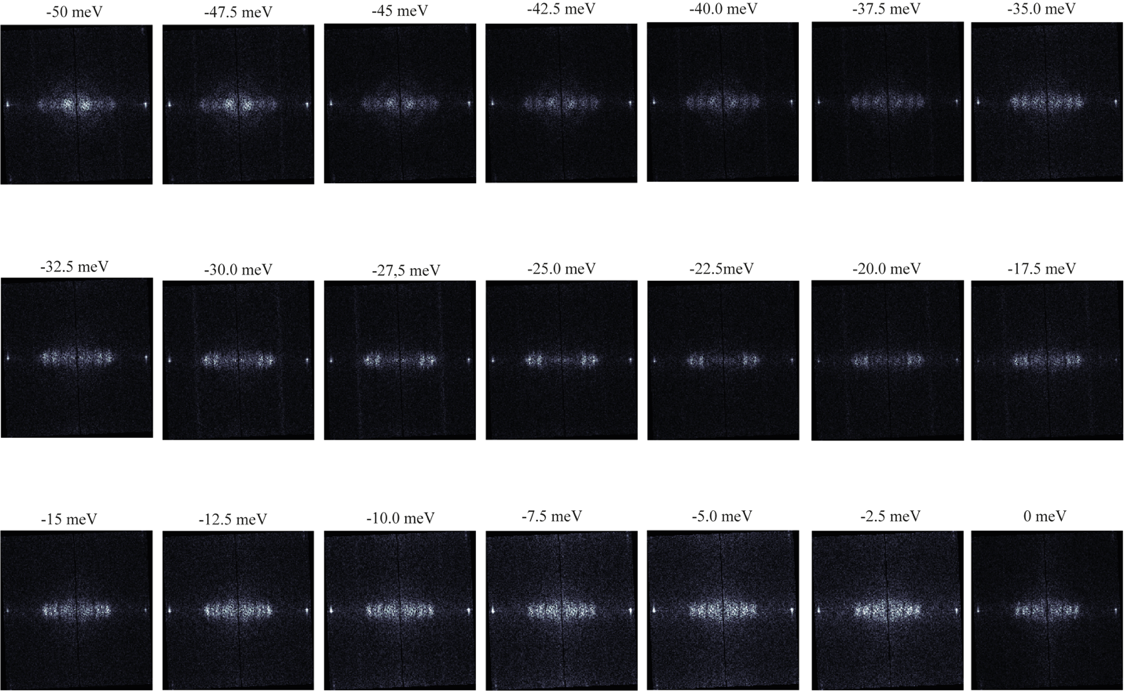


Figure S3 – Additional non-symmetrized QPI data acquired on an AFM surface. The FFTs show the C_2_ symmetric structure, with no QPI signature in the direction perpendicular to the Q1, Q2, and Q3 vectors as defined in the main text. Data was acquired using a Cr tip.


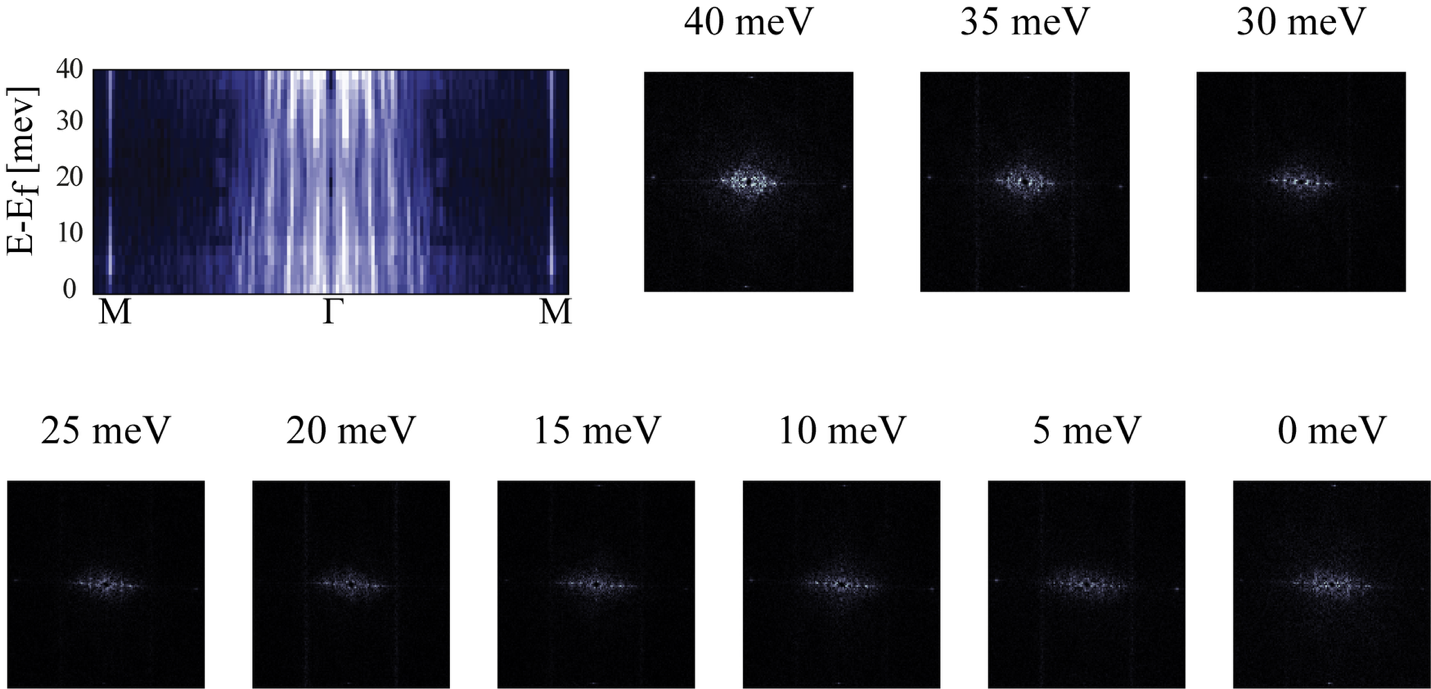


Figure S4 – Additional non-symmetrized QPI data acquired on an AFM surface above the Fermi level. The FFTs show the C_2_ symmetric structure, with no QPI signature in the direction perpendicular to the Q1, Q2, and Q3 vectors as defined in the main text. Data was acquired using a Cr tip.

**JDOS simulation including surface Dirac cones:**


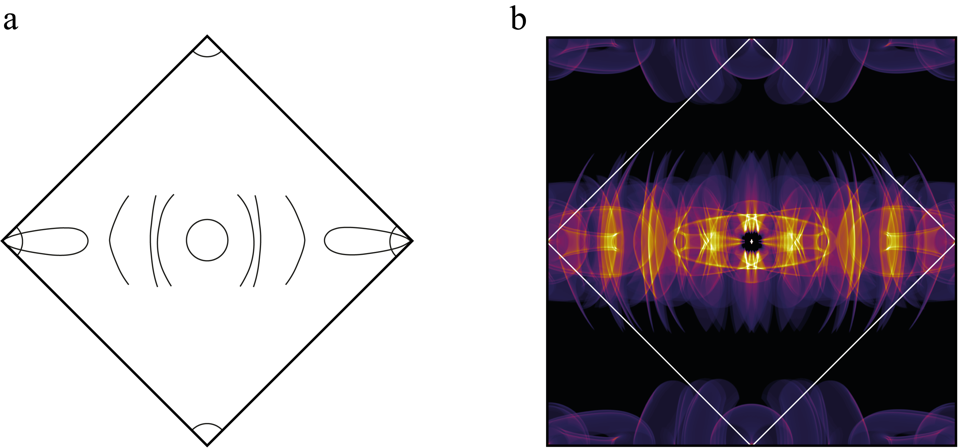


Figure S5 – JDOS simulation of the QPI including the Dirac cones. Spin texture has not been considered. **a** Cartoon depicting the electron and hole like surface states at E_F_, including the Dirac cones at the Γ and M points of the Brillouin zone. **b** Calculated FFT of the QPI patterns for the surface Dirac cones, electron like surface states and Fermi arcs in a single AFM domain. White square represents the Brillouin zone. Due to Dirac cones at the M point a replica of the original C_2_ symmetric scattering vectors appear, which is missing in our data.

**QPI suppression below *E*=-120 meV on the AFM termination:**


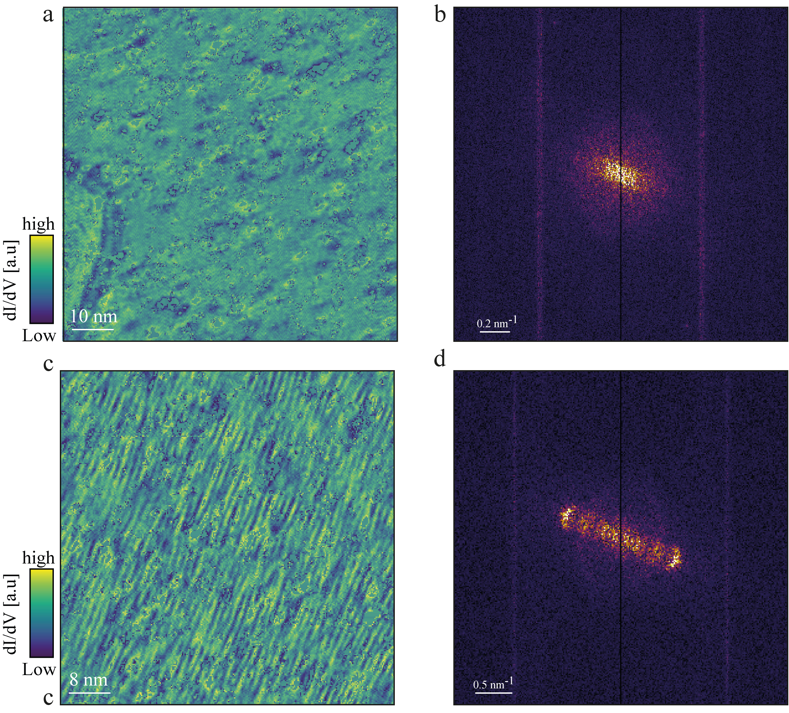


Figure S6 – Conductance maps at different energies, below and at the surface states energies (as seen in ARPES). **a** E= -120 meV slice from a dI/dV map taken with a W tip showing no QPI patterns, and no wave like signature, I_s_ = 450 pA, V_s_ = -120 meV, V_mod_ = 6 meV, f = 907.5 Hz. **b** FFT of the dI/dV map at **a**, with the QPI missing. **c** E = -40 meV slice from a dI/dV map taken with a W tip and the same location as **a** showing the QPI patterns with the wave like signature, I_t_ = 240 pA, V_s_ = -40 meV, V_mod_ = 3 meV, f = 907.5 Hz. **d** FFT of the dI/dV map at **c** showing a clear C_2_ symmetric QPI signal. Data was acquired using a W tip.

**QPI on AFM termination showing disappearance in the paramagnetic phase, *T* > *T*_Néel_:**


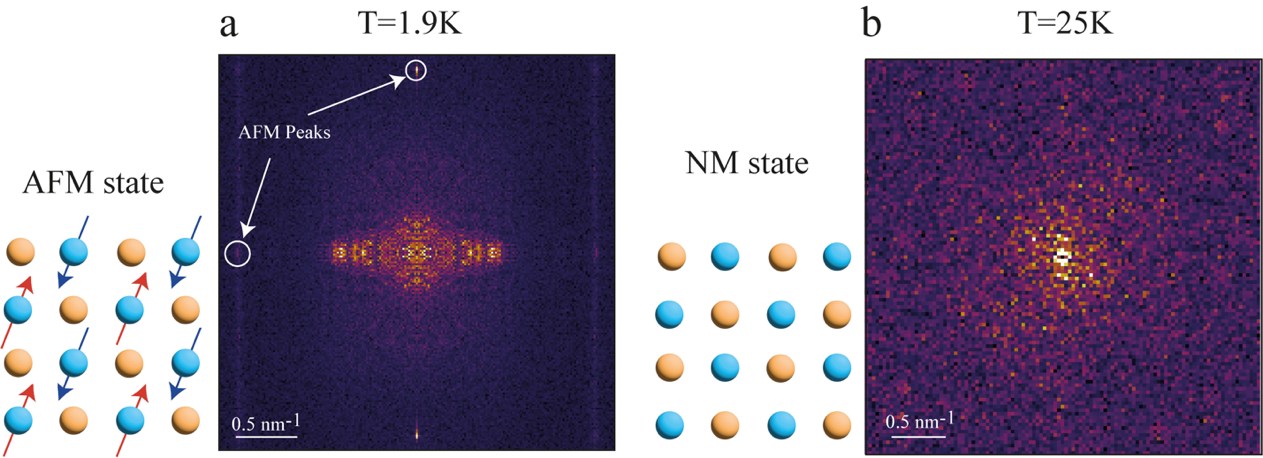


Figure S7 – Evolution of the C_2_ symmetric QPI with temperature at E_F_. **a** FFT of the slice at E_f_ of a conductance map taken at *T*=1.9K < *T*_Néel_. AFM peaks are visible at q corresponding to an AFM vector of 0.64 nm. Small panel: cartoon depicting the AFM surface, with the spin and AFM vector in plane. **b** Same as **a** at *T*=25K > *T*_Néel_, and at the same location, with the AFM peaks missing and no QPI. Small panel: cartoon depicting the non-magnetic surface. The QPI are clearly absent in this map, due to the disappearance of the surface states above Néel temperature. *V_s_* = -40 meV, *I_t_* = 130 pA, *V_mod_* = 6 meV, *f* = 907.5 Hz. Panel **a** show the raw data without cleaning the noise in the data which results in weak vertical lines on each side of the FFT. Such noise originates from system conditions and does not reflect on the existence of QPI signal. Data was acquired using a Cr tip.

**Absence of QPI signal on the FM surface termination:**


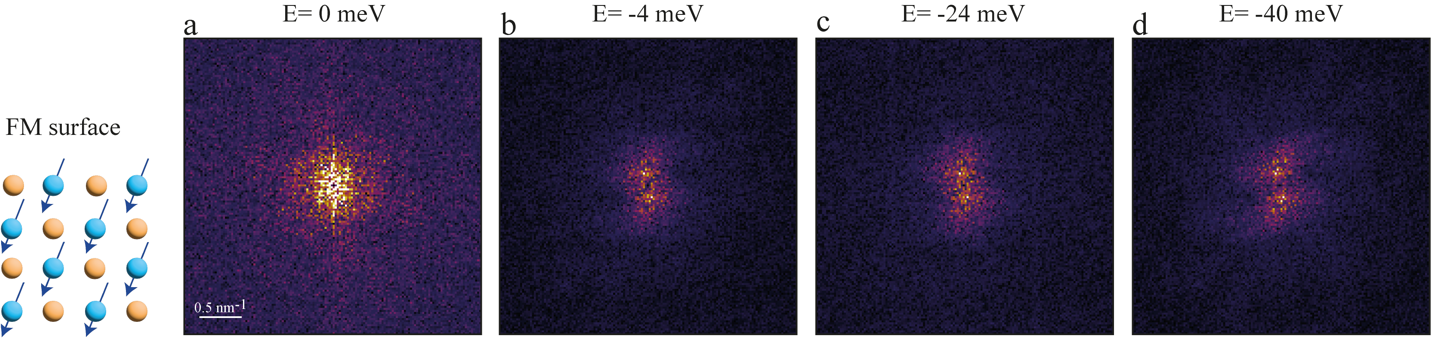


Figure S8 - FFT of conductance maps of different energies along the surface states dispersion taken on a FM surface at *T*=1.9K<*T*_Néel_. The AFM peaks are missing, together with the QPI, in **a** 0 meV, **b** -4 meV, **c** -24 meV and **d** -40 meV, as opposed to the corresponding maps taken on the AFM surfaces (supplementary Figure S2). QPI signal is absent due to the disappearance of the surface states as explained in the main text. *V_s_* = -40 meV, *I_t_* = 200 pA, *V_mod_* = 5 meV, *f* = 907.5 Hz. Data was acquired using a Cr tip.

**Gaussian-decay fit of the conductance as function of distance from the edge:**


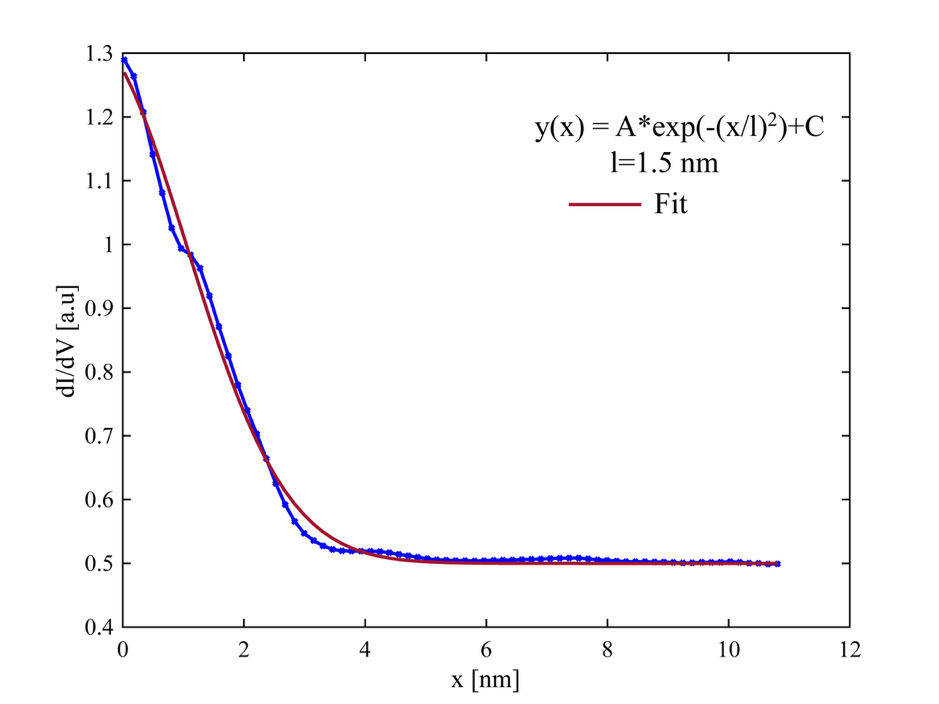


Figure S9 – Fit of the conductance as function of distance from the edge, at bias energy of E=100 meV to a Gaussian decay. The decay length scale (l) is in excellent agreement the one expected from the behavior of an edge state in the Bernevig-Huges-Zhang model in the quantum spin hall phase. The data used is the integration of the data parallel to the edge as shown in Figure 3.

**Additional data showing step edge states on FM step edges**

**
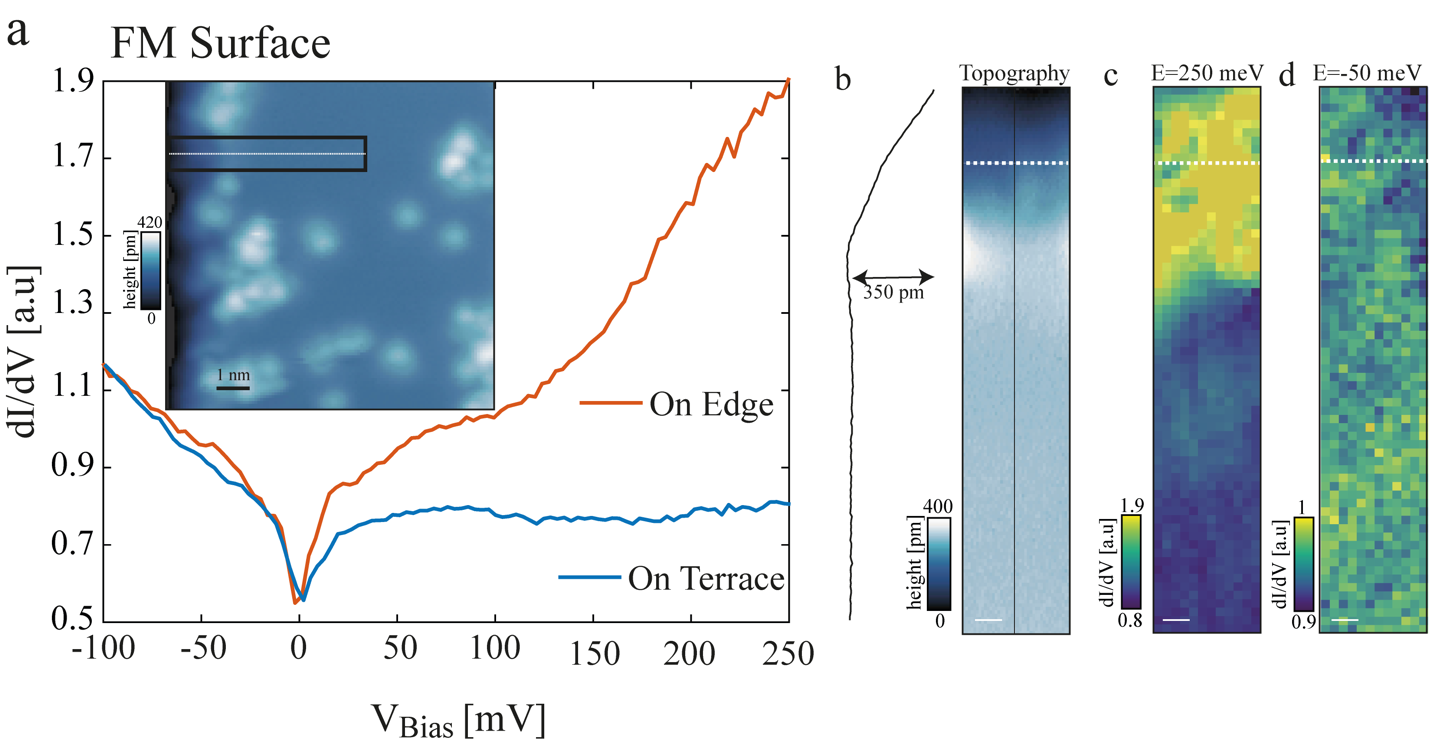
**

Figure S10 – Additional step edge data. **a** dI/dV spectra acquired on a terrace and on a step edge of a FM surface. An average taken on the edge (orange curve) shows the increased conductance on the step edge relative to the average taken on the terrace (blue curve). Inset: Large scale topography taken on the terrace. White dashed line marks the linecut along which the data of FigS10 was acquired. **b** Smaller topography taken on the step edge and the line profile obtained at the solid vertical black line. V_s_ = -100 meV, I_t_ = 150 pA. **c** dI/dV map at E = 250 meV on the same area showing the edge state which is localized within 1.5 nm of the edge. **d** dI/dV map at E = -50 meV. Dashed white lines mark the location of the edge in all panels. All scale bars in b-d are 0.25 nm. Data was acquired using a Cr tip.

**Edge state and QPI measurements using a W tip:**

As shown below, edge states can be seen in data obtained with W-tips. Here we use the distinct QPI fingerprints to identify the FM and AFM surface terminations. Consistent with data obtained with Cr tips, clear step edge states are visible on the FM step edges and absent on the AFM edges.

**
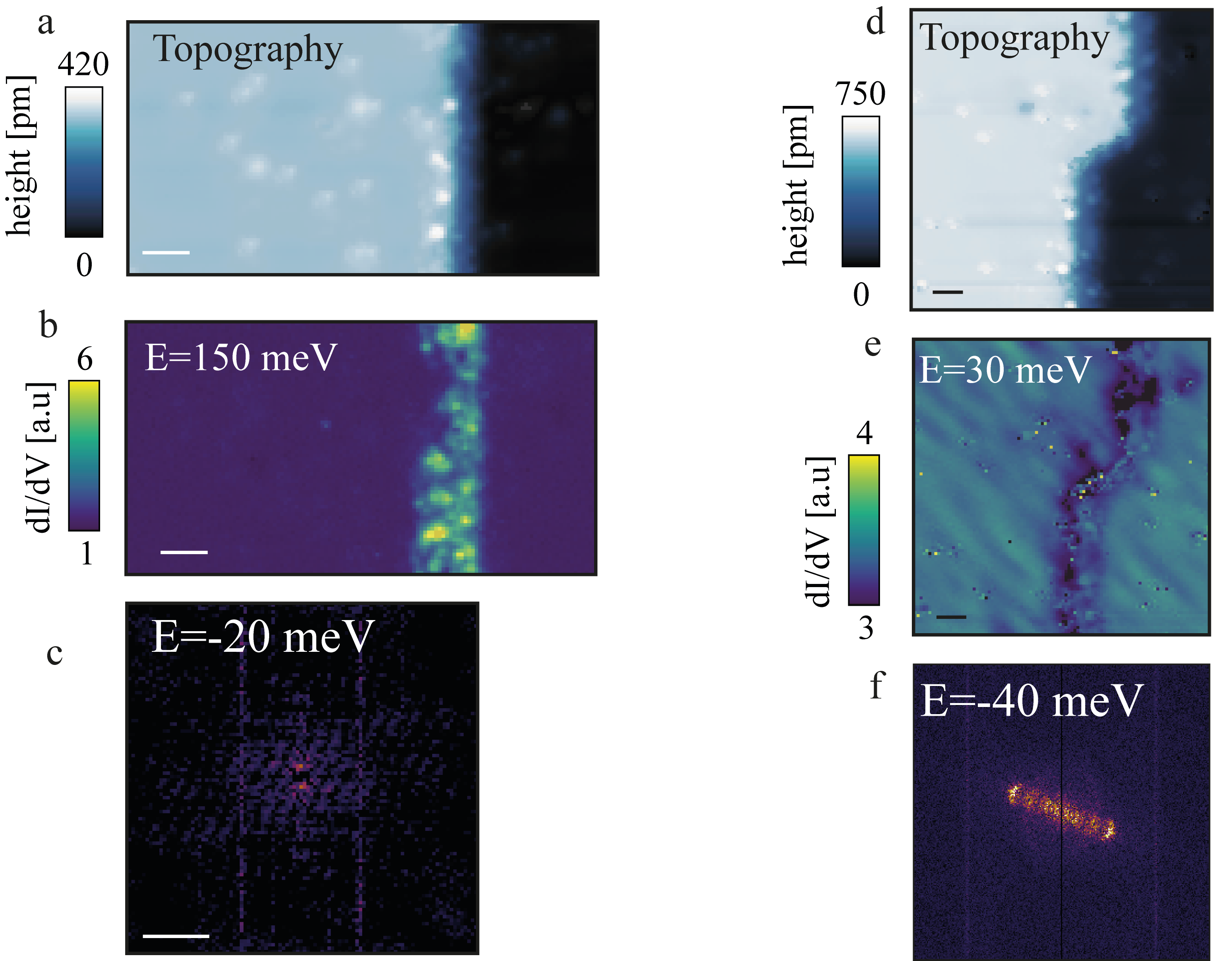
**

Figure S11 – *dI/dV* maps on a FM and AFM surfaces with step edges. **a** Topographic image of a single unit cell step edge from a FM surface to a FM surface. **b** *dI/dV* conductance map measured in the same location as **a** showing excess density of states localized on the edge, corresponding to an edge state, scale bar in a-c is 2.5 nm. **c** No QPI is observed at a bias where clear QPI exists on AFM surfaces. This confirms the surface identification as a FM termination. *V_s_* = -65 meV, *I_t_* = 250 pA*.* *V_mod_* = 5 meV. Scale bar is 0.65 nm^-1^ **d** Topographic image of an edge on the AFM surface. **e** *dI/dV* conductance map measured in the same location as **d**. The edge state is absent on the edge with a clear QPI signal seen revealing it to be an AFM surface, scale bar in d and e is 2 nm. *V_s_* = 30 meV, *I_t_* = 150 pA. **f** FFT of the QPI pattern of the same terrace, also given in Supplementary Figure S4. The FFT shows a clear C_2_ symmetric QPI signal. Data was acquired using a W tip

**Spectra on AFM termination showing the absence of the edge state below *T*_Néel_:**


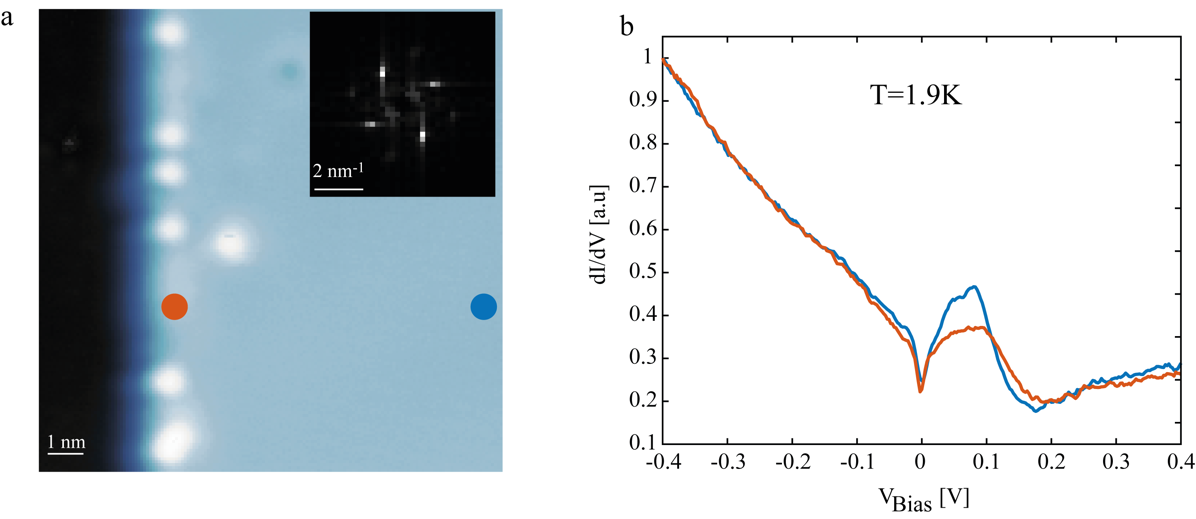

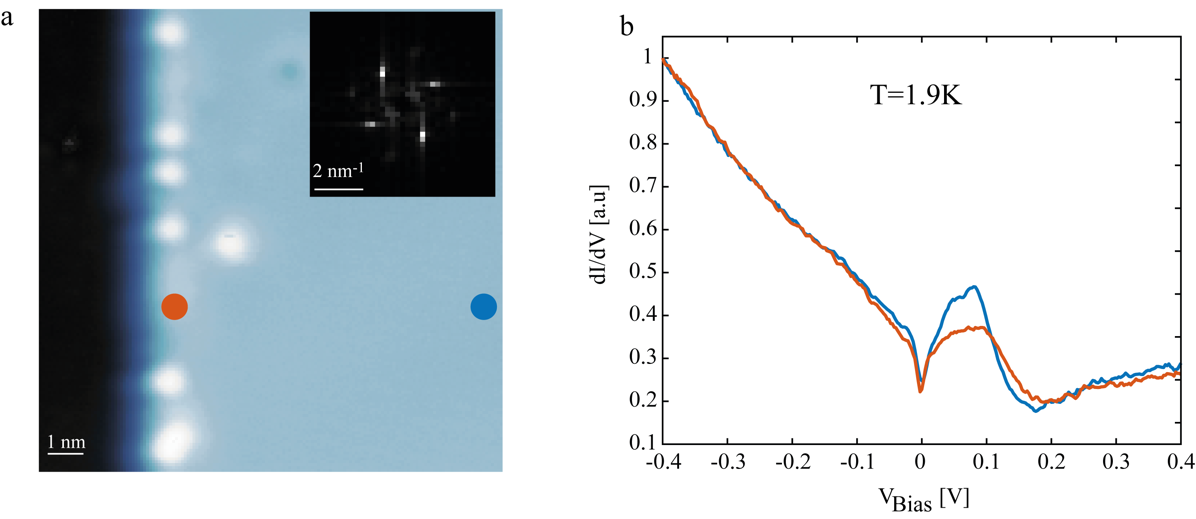


Figure S12– Absence of an edge state on the AFM surface. **a** Topographic image of a single unit cell step edge from an AFM surface to an AFM surface. white dots are adatoms on the surface. inset: FFT showing the AFM peaks taken on the same layer adjacent to the edge. **b** *dI/dV* spectra locally measured in a region on the step edge (orange) and a few nm away from it (blue). The spectrum on the edge (orange) shows no enhancement of density of states that would be expected for an edge mode, and in fact shows a small suppression *V_s_* = -400 meV, *I_t_* = 500 pA, *V_mod_* = 4 mV*.* Data was acquired using a Cr tip.

**Adatoms spectra:**


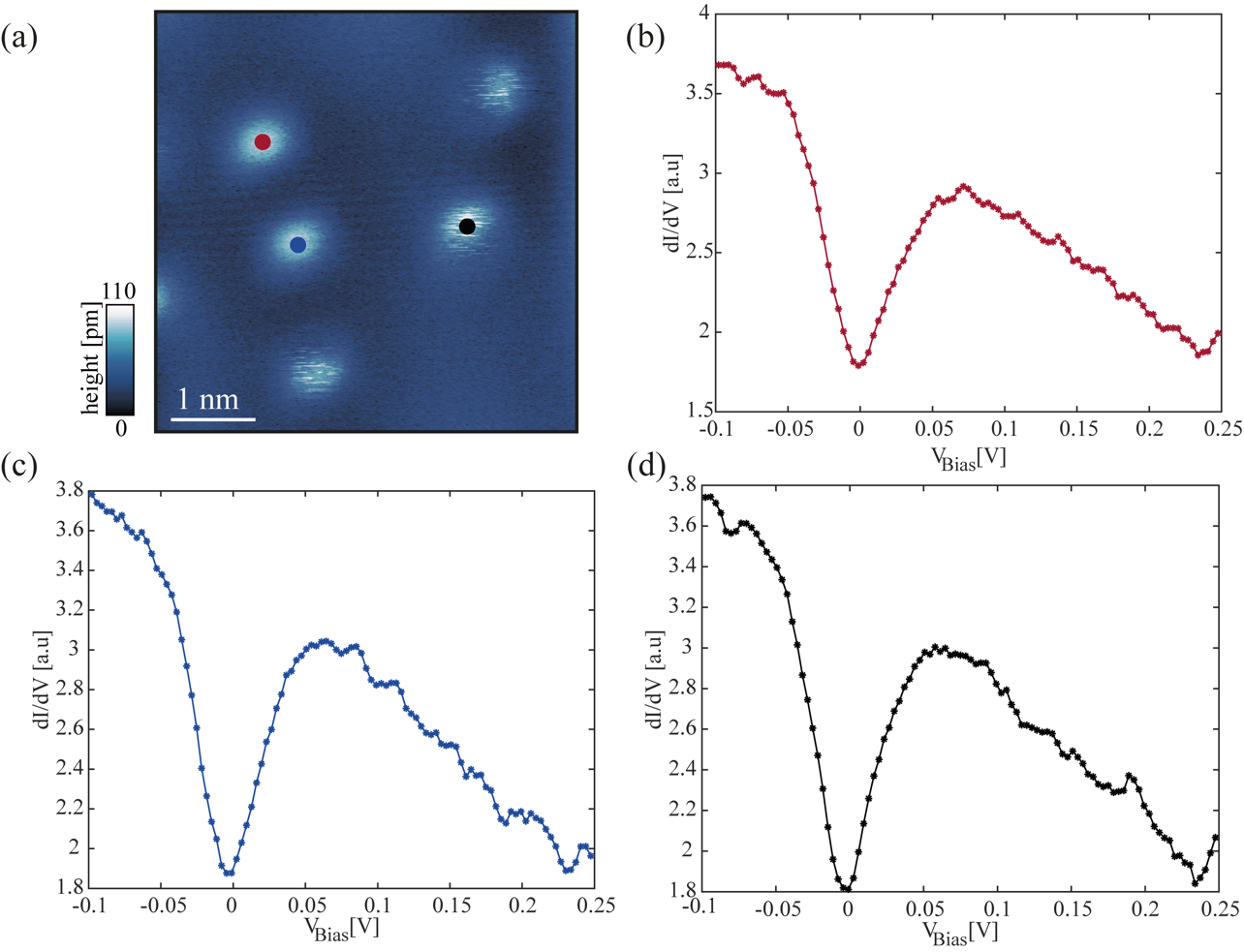


Figure S13 – Spectra on adatoms. **a.** Topographic scan taken in a FM surface together with adatoms. V_s_ = -100 mV, I_t_ = 150 pA. **b-d.** Spectra taken on individual adatoms as marked in a using the respective color for each spectrum. No enhancement is seen for energies above the Fermi level, which indicates the edge state could not be explained as simple consequence of adatoms on the surface. For all spectra shown: V_s_ = -100 mV, I_t_ = 100 pA, V_mod_ = 4 mV. Data was acquired using a Cr tip.

**Band structure extracted from DFT:**

*
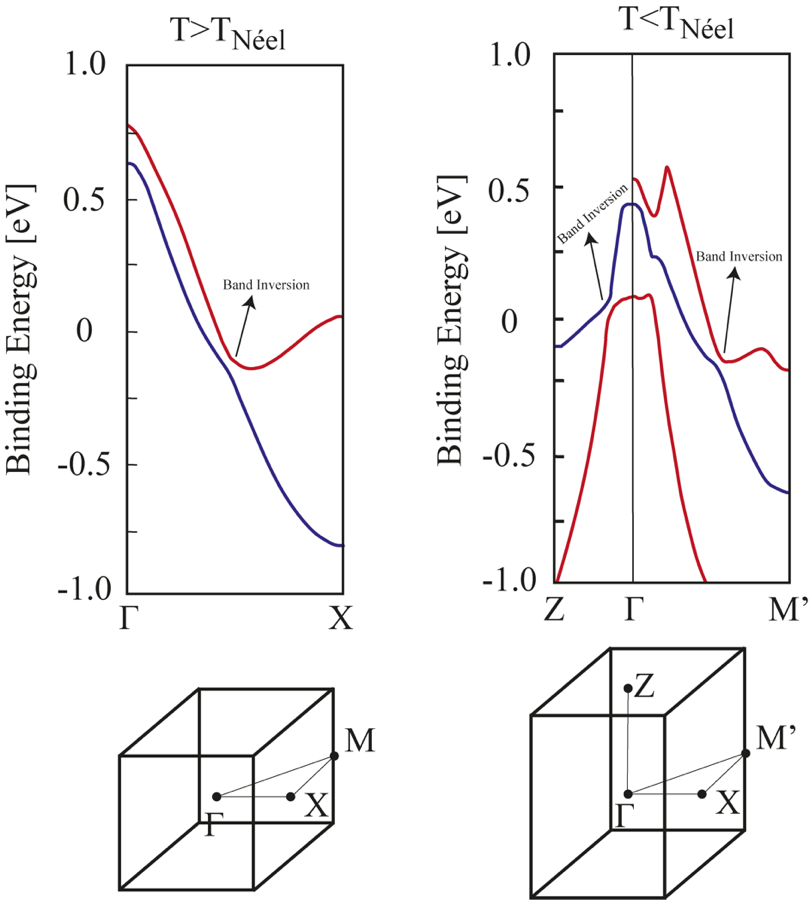
*

Figure S14 – Band dispersion of the bands around the Fermi level above (left) and below (right) T_Néel_. The dispersion is for the FM surface in the AFM phase showing the band inversion gap is below and above E_F_ for the Γ-M’ line and completely above E_F_ along Γ-Z. In the paramagnetic phase the band inversion is completely bellow the Fermi level. Data is taken from ref. 29, using the same notations for the high symmetry lines. Lower part shows the Brillouin zones in each phase, note that due to rotation of the unit cell the new M’ direction in the AFM phase is along the X direction of the paramagnetic phase.
